# Validity of C-reactive protein (CRP) for diagnosis of neonatal sepsis

**DOI:** 10.12669/pjms.313.6668

**Published:** 2015

**Authors:** Effat Hisamuddin, Aliya Hisam, Sughra Wahid, Ghulam Raza

**Affiliations:** 1 Effat Hisamuddin, MBBS, FCPS (Paeds), Consultant Paediatrician, Tehsil Headquarter Hospital (THQ), Chakdara, Khyber Pakhtunkhwa, Pakistan; 2 Aliya Hisam, MBBS, MPH, Assistant Professor, Community Medicine Department, National University of Sciences and Technology (NUST), Army Medical College, Rawalpindi, Pakistan; 3 Sughra Wahid, MBBS, FCPS (Paeds), MRCPCH, Head of Pediatric Department, Kahuta Research Laboratories (KRL) Hospital, G9/1, Islamabad, Pakistan; 4 Ghulam Raza, MBBS, MSc (Medical Administration), Vice Principal, Army Medical College, Rawalpindi, Pakistan

**Keywords:** C-reactive protein, Neonates, Neonatal Sepsis

## Abstract

**Objective:**

To determine the validity of C-reactive protein levels for diagnosis of neonatal sepsis.

**Methods:**

A cross sectional (Validation) study was conducted at Neonatology unit in KRL general hospital (emergency/OPD) of 7 months duration from February 2012 to August 2012. By using purposive sampling technique, 147, sample size was calculated by using WHO sample size calculator taking sensitivity 75%, specificity 95%, expected prevalence 50%, desired precision 10% and confidence level 95%.

**Results:**

Mean age of the neonates was 5.72 days + 3.86. Male patients were 81(55.1%) while 66(44.9%) were female. Neonatal sepsis was observed in 43(29.25%) and were confirmed through blood culture while 104(70.75%) were not confirmed on blood culture as neonatal sepsis. The sensitivity and specificity of CRP in diagnosis of acute neonatal sepsis was 76.92% and 53.49% respectively while it had a positive predictive value of 80% and negative predictive value of 48.94%. Over all the diagnostic accuracy of CRP in diagnosis of neonatal sepsis was 70.07%.

**Conclusion:**

CRP estimation does have a role in the diagnosis of neonatal sepsis but the test is not specific enough to be relied upon as the only indicator.

## INTRODUCTION

Septicaemia is a recognised cause of morbidity and mortality in the new-borns in the developing countries.^1^ Septicaemia is well-defined as a “clinical syndrome characterized by systemic signs/symptoms and bacteraemia during the 1^st^ month of life”. Septicaemia is known as ‘early onset’ disease if present during 5-7 days of life and considered as ‘late onset’ if it follows after the first week.^2^

Neonatal septicemia is a serious illness but curable if identified early. The primary threatening signs and symptoms are mostly nonspecific and can easily be mixed up with the non-infective causes. Nonspecific signs/symptoms makes it very challenging to formulate a timely clinical diagnosis.^3^ Neonatal physician after evaluating many test are looking for a test that would help in neonatal sepsis diagnosis and also quickly confirms it and also that decisively rules it out.^4^ Diagnostic test like blood cultures are time consuming so correct diagnosis gets delayed and problematic. Cultural proven sepsis is about 2 per 1000 live birth. Seven to thirteen percent neonates are assessed for neonatal sepsis and out of them only 3 to 8 percent show culture proven sepsis.^5^ CRP along with some test (TLC, ANC, and thrombocytopenia) are very sensitive in detecting negative cases of neonatal sepsis.^6^

A new born will suffer if its infection is not diagnosed properly: under diagnosed or over diagnosed.^7^ For early diagnosis of newborn sepsis, combined and/or alone interleukin-6 (IL-6) and C-reactive protein (CRP), has a recognized role.^8^ CRP serial measurement in infection progress is helpful, also in infection diagnosis. The negative predictive value, positive predictive value, sensitivity and specificity in infants (term and preterm) were observed to be 99.0%, 97.8%, 61.5% and 75.0% respectively.^9^

C-reactive protein was first described by Tillet and Francis in 1930. They concluded that it is a protein that helps in complement binding to foreign or damaged cells in response to inflammation and rising to peak levels after fifty hours.^10^ CRP production is a non-specific response to disease and cannot be used alone as a diagnostic test for septicaemia. Along with clinical evidence of the disease, CRP provides good idea regarding septicaemia diagnosis.^9^

This study aimed to examine the part of CRP in neonatal sepsis to see if it can be used as a tool to find the time period when antibiotics treatment can be safely discontinued in case of suspected neonatal septicaemia. In many centers only one CRP test is done on admission and if it comes out to be negative antibiotics are stopped and later on baby comes back with severe sepsis. This is because liver usually takes about 8 to 9 hours for CRP production and in next 48 to 72 hours CRP reaches peak levels. Few studies have been done on validity of quantitative analysis of CRP test but in many centers only qualitative test of CRP is available which is cheap. Checking the validity of qualitative CRP test when done twice, one at admission and second at 72 hours after the first one is very essential.

## METHODS

A cross sectional (Validation) study was conducted at KRL General Hospital (emergency/OPD) of 7 months duration from February 2012 to August 2012. About 147 sample size was calculated by using WHO sample size calculator taking sensitivity 75%^9^, specificity 95%^11^, expected prevalence 50%, desired precision 10% and confidence level 95%. After taking informed and written consent from parents and permission from KRL hospital ethical committee, neonates brought to neonatology unit were selected by purposive sampling technique. All babies from 0 to 28 days of life having suspected neonatal sepsis were included in the study. Suspected neonatal sepsis was considered if neonate had clinic pathological features of perinatal risk factors i.e. maternal pyrexia (within 1 week prenatal and/or 48 hours postnatal), prolonged rupture of membranes (18 hours), foul smelling vaginal discharge or/and maternal urinary tract infection diagnosed in last month. Neonates having unexplained hypothermia/hyperthermia, lethargy, irritability, poor feeding or milk intolerance, respiratory dysfunction evidenced by apnea (>10sec.), tachypnoea (>60 breaths/minute), cardiovascular dysfunction such as persistent tachycardia (>160 beat/min) or bradicardia (<100 beats/min), poor peripheral circulation, hypotonia or circumoral cyanosis or pallor were also included. Baby who had suffered birth asphyxia, very low birth weight <1500 grams, extremely premature <32 weeks gestation and neonates who already had taken antibiotics were excluded from the study.

All patients included in the study were started on empirical antibiotics after drawing samples for blood cultures and CRP was sent to laboratory. Strict aseptic measures were taken to rule out any systemic bias while taking blood cultures. A second sample for determination of CRP was drawn 72 hours after the first one. Two CRP samples were taken, one at the time of admission and second one at 72 hours after the first one. CRP were read as negative when level was less than 5mg/dl and positive when level was more than 5mg/dl. Blood culture was followed for growth up to 7 days. The results of the CRP were verified by laboratory technician and head of pathology department. Data collection tool was a pre-tested performa. Suspected neonatal sepsis patients were started on empirical antibiotic therapy on admission and first CRP and blood culture were sent for analysis. If the first CRP came negative, the antibiotic therapy was continued and if the second CRP was also negative, the antibiotic therapy was discontinued. But if the second CRP came positive, the antibiotic therapy was continued or changed, looking at the clinic pathological picture of the patient. If first and second CRP both were positive, the therapy was continued and the culture and sensitivity report were awaited for making decision regarding the antibiotic therapy.

SPSS version 20 was used for statistical analysis of the collected data. Mean and standard deviation were calculated for numerical variables i.e. age and weight of baby. Frequency and percentages were presented for categorical variables i.e. qualitative CRP and blood culture results. Sensitivity, specificity, negative and positive predictive values for CRP in identifying babies with culture proven neonatal sepsis were also calculated.

## RESULTS

In this study, 147 patients with suspected neonatal sepsis were inducted. Males were 81(55.1%) of while 66(44.9%) were female patients. Male to female ratio 1.22:1.

Neonatal age was divided in four categories out of which most presented in young age i.e. less than or equal to 5 days which was 91(61.9%). About 42(28.6%) patients were of age 6-10 days, 7(4.8%) were of age range 11-15 days and 7(4.8%) presented at age more than 15 days. The study included age ranged from 1 up to 27 days. Mean age was 5.72 days ± 3.86.

Age wise distribution of results shows that 66(63.5%) neonates having sepsis were found to be in less than or equal to 5 days of age. Twenty seven (26%) neonates having sepsis had age range of 6-10 days, 6(5.8%) had age range of 11-15 days and 5(4.8%) had age range of more than 16 days. Similarly 25(58.1%) neonates had age less than or equal to 5 days, 15(34.9%) had age 6-10 days, 1(2.3%) neonates had age 11-15 days and 2(4.7%) neonates had more than 15 days of age were observed as not having neonatal sepsis, as shown in Table-I.

**Table-I:** Gender and age wise distribution of culture results (n=147).

Characteristics	Neonatal Sepsis on Culture	
	Yes	No
*Gender*		
• Female	43 (41.3%)	23 (53.5%)
• Male	61 (58.7%)	20 (46.5%)
*Patient Age (in days)*		
• <= 5	66 (63.5%)	25 (58.1%)
• 6 - 10	27 (26.0%)	15 (34.9%)
• 11 - 15	6 (5.8%)	1 (2.3%)
• 16+	5 (4.8%)	2 (4.7%)

Gender wise distribution of neonatal sepsis on culture results showed that the males were more exposed as compared to females i.e. 58.7% and 41.3% respectively. Neonatal sepsis was confirmed in 104 (70.75%) while 43(29.25%) were not confirmed through culture reports, details are shown in Table-II.

**Table-II:** Accuracy of CRP in diagnosis of neonatal sepsis (n=147).

	Culture sensitivity		
CRP levels at 72 hrs	Positive	Negative	Total
Positive	80	20	100
Negative	24	23	47
Total	104	43	147

CRP done at the time of admission was 94(63.9%) positive and 53(36.1%) negative while CRP done after 72 hours of the first one were 100(68%) positive and 47(32%) negative. The sensitivity and specificity of CRP (at 72 hours of admission) in diagnosis of acute neonatal sepsis were 76.92% and 53.49% respectively while it had a positive predictive value of 80% and negative predictive value of 48.94%. Over all the diagnostic accuracy of CRP in diagnosis of neonatal sepsis was 70.07%, as shown in Table-III.

**Table-III:** Validity and predictive outcomes of CRP.

• Sensitivity	76.92%
• Specificity	53.49%
• Positive Predictive value	80%
• Negative predictive value	48.94%
• Accuracy	70.07%

## DISCUSSION

Neonatal sepsis is the major and common cause of morbidity and mortality. The incidence is much higher in the developing world. Early diagnosis and effective treatment is the best way to reduce morbidity and mortality. The delay in diagnosis and initiating therapy are the main reasons for high mortality. Blood culture is still regarded as a gold standard for diagnosis. Different hematologic parameters, multiple inflammatory cytokines and acute phase reactants levels are used in this regard. Among the various tests CRP role in neonatal sepsis has been vastly studied.

In this study, validity of CRP in the diagnosis of sepsis was studied on 147 neonates. One hundred and four cases of neonatal sepsis confirmed on blood culture were evaluated. Most of the patients evaluated had the known risk factors and clinical features associated with sepsis. According to one study, CRP had the sensitivity and specificity of 58.33% and 56.52% respectively. The test had a positive predictive value of 67.74% and 48.27%.^2^

Benitz and colleagues^12^ have reported that the sensitivity of the test is only 40% if performed at presentation. There is generally a delay of up to 24 hours between the onset of symptoms of infection and rise in serum CRP. The sensitivity is increased upto 90% if performed 24 hours later. The same was observed in a study by Mather NJ and colleagues^13^ showing a sensitivity rise from 22% to 61% with increasing time after admission. Wagle S and Colleagues^14^ studied the role of CRP in sepsis in very immature babies and documented that the sensitivity/specificity of CRP on Day 1 was 62% and 87.7% increasing upto 70.2 and 97% on Day 2. One of our limitations was that we only recorded positive and negative result of the CRP result rather than measuring the exact value. So we cannot comment on the rising titer of the CRP in neonatal sepsis.

Chan DK and colleagues^15^ gave a cutoff CRP level of 7 mg/L. The sensitivity, specificity, negative and positive predictive values were 56%, 72%, 71% and 57% respectively. In our study CRP was found positive in 46.5% of culture negative cases, while it was negative in 23.7% of culture proven sepsis. In 3 culture proven cases, CRP was found positive at 0 hrs and its level raised at 72 hours detection in spite of empirical antibiotic treatment. Clinically the condition of neonates also deteriorated, end result was fulminant sepsis in two cases.

Jave DL^16^ stated that monitoring of CRP over time may be used to in determining the response of the treatment after the primary diagnosis. They were discharged home after 5 days of intravenous antibiotic therapy. Jin Cherdze and colleagues^17^ concluded in their study that quantitative CRP is a rapid, sensitive diagnostic marker for identification of sepsis in preterm infants.^17^ In our study, we also found CRP a good indicator of neonatal sepsis as qualitative status of CRP helped in identification of neonatal sepsis and also in deciding the line of management of the patient.

Keeping in view the mortality associated with neonatal sepsis, treatment is often initiated on suspicion of sepsis. In this study CRP was found positive in 20 infants in culture negative cases. This could be due to the administration of intrapartum antibiotics, influencing the result of culture. These neonates cannot be excluded from the study because fatal infection has been reported in the presence of a negative blood culture.^4^ Similarly infants with intrapartum risk factors (augmentation of labor using oxytocin, epidural anesthesia, maternal pyrexia and meconium stained liquor) and clinical features of sepsis were also included. Raised CRP levels are found in 50-90% of neonates from six hours of onset of bacteremia. Raised levels are not specific for bacterial infection.^18^ Other conditions in which CRP levels are raised are asphyxia, shock, intraventricular haemorrhage, surgery and meconium aspiration.^19^,^20^

Latex agglutination slide test was used for the detection of CRP in the study. It is an easy to perform, economical and readily available method. Other technique available is the quantitative radioimmuno diffusion technique. It is more specific but costly and time consuming. As per results of the study, CRP cannot be regarded as a good screening test for early diagnosis of sepsis but can be made part of a scoring system. Hematologic parameters along with clinical criteria should also be included in this scoring system. This would decrease the indiscriminate use of antibiotic on one hand and reduce the delay in initiation of therapy on the other. Manucha and colleagues^21^ mentioned in their study the importance of a scoring system. They evaluated the scoring system designed by Rodwell et al.^22^ Ahmed Z and colleagues^6^ evaluated the role of CRP as a diagnostic marker in combination with hematological parameters.

Considering all these studies and the results of our study, a scoring system can be formulated by panel of experts for the detection of neonatal sepsis. Scoring system should include other simple to perform and economical tests besides CRP, thus enabling neonatal sepsis early detection.

## CONCLUSIONS

CRP estimation does have a role in the diagnosis of neonatal sepsis but the test is not specific enough to be relied upon as the only indicator. The sensitivity, specificity, positive and negative predictive values as calculated in this study are not high enough to make it a good screening test. Considering the high morbidity and mortality associated with it, clinical criteria along with other hematological parameters and diagnostic markers along with serial CRP should be considered in evaluating a neonate for sepsis.
